# WRKY transcription factors (TFs): Molecular switches to regulate drought, temperature, and salinity stresses in plants

**DOI:** 10.3389/fpls.2022.1039329

**Published:** 2022-11-08

**Authors:** Muneer Ahmed Khoso, Amjad Hussain, Faujiah Nurhasanah Ritonga, Qurban Ali, Muhammed Malook Channa, Rana M. Alshegaihi, Qinglin Meng, Musrat Ali, Wajid Zaman, Rahim Dad Brohi, Fen Liu, Hakim Manghwar

**Affiliations:** ^1^ Lushan Botanical Garden, Chinese Academy of Sciences, Jiujiang, Jiangxi, China; ^2^ Department of Life Science, Key Laboratory of Saline-alkali Vegetation Ecology Restoration, Ministry of Education, Northeast Forestry University, Harbin, China; ^3^ College of Plant Science and Technology, National Key Laboratory of Crop Genetic Improvement, Huazhong Agricultural University, Wuhan, Hubei, China; ^4^ State Key Laboratory of Tree Genetics and Breeding, Northeast Forestry University, Harbin, China; ^5^ Department of Plant Pathology, College of Plant Protection, Nanjing Agricultural University, Key Laboratory of Monitoring and Management of Crop Diseases and Pest Insects, Ministry of Education, Nanjing, China; ^6^ Biology Section, Nobles International School, Jeddah, Saudi Arabia; ^7^ Department of Biology, College of Science, University of Jeddah, Jeddah, Saudi Arabia; ^8^ Department of Biology and Food Engineering, Bozhou University, Bozhou, China; ^9^ Department of Plant Sciences, Faculty of Biological Sciences, Quaid-i-Azam University Islamabad Pakistan, Islamabad, Pakistan; ^10^ Department of Life Sciences, Yeungnam University, Gyeongsan, South Korea; ^11^ Department of Animal Reproduction/Theriogenology, Faculty of Veterinary Science, Shaheed Benazir Bhutto University of Veterinary and Animal Sciences, Sakrand, Pakistan

**Keywords:** WRKY TFs, drought-stress, salinity-stress, temperature-stress, cold-stress, plant development and growth, plants/crops

## Abstract

The WRKY transcription factor (TF) belongs to one of the major plant protein superfamilies. The WRKY TF gene family plays an important role in the regulation of transcriptional reprogramming associated with plant stress responses. Change in the expression patterns of WRKY genes or the modifications in their action; participate in the elaboration of numerous signaling pathways and regulatory networks. WRKY proteins contribute to plant growth, for example, gamete formation, seed germination, post-germination growth, stem elongation, root hair growth, leaf senescence, flowering time, and plant height. Moreover, they play a key role in many types of environmental signals, including drought, temperature, salinity, cold, and biotic stresses. This review summarizes the current progress made in unraveling the functions of numerous WRKY TFs under drought, salinity, temperature, and cold stresses as well as their role in plant growth and development.

## Introduction

The WRKY family is a group of transcription factors (TFs) that are widely distributed in plants and play important roles in plant growth and development, and biotic and abiotic stress management. The increased exposure in plants to various stresses, such as extreme temperatures, drought, and salinity is a global threat to key crops which significantly affect plant/crop growth and productivity. Many TF genes help plants withstand to adverse conditions and remain potential genomic candidates for widespread use in crop breeding. WRKY TFs represent important molecular switches that evaluate plant development processes and are involved in regulating responses to various stresses. Under stress conditions, plants can initiate a variety of changes at the molecular, cellular, and physiological levels, including stomatal closure, reduced photosynthesis, higher osmolality accumulation, and induction of many stress response genes (Shinozaki and Yamaguchi-Shinozaki, 2007; Masclaux-Daubresse et al., 2010; Kapoor et al., 2020). Genetic engineering is considered an alternative to increasing stress tolerance and has made significant contributions to changing the agronomic properties of crops. Many genes encoding functional proteins, TFs, and proteins involved in signal transduction pathways have been identified as genes responding to abiotic stresses (Turan et al., 2012; Rashid et al., 2020; Cohen et al., 2021). Many TF families, such as WRKY, AP2 (APETLA2)/ERF (ethylene responsive factor), and NAC (NAM, ATAF1/3, and CUC1/2), are unique to plants and have important and specific functions (Jiang et al., 2017).

## Structural features and homology of the WRKY TFs

WRKY protein have the unaltered sequence *WRKYGQK* (hence called WRKY) and a 60 amino acid DNA binding domain comprising a zinc finger-like domain (CX7CX23HXC or CX4-5CX22-23HXH) (Rushton et al., 1996; Finatto et al., 2018). WRKY TFs are classified into different groups; several WRKY proteins are placed in group I, containing two WRKY domains. WRKY proteins comprising one WRKY domain and a Cys2-His2 zinc finger motif are placed in group II. Furthermore, based on additional structural motifs maintained outside the WRKY domain, group II is subdivided into five subgroups (group IIa, group IIb, group IIc, group IId, and group IIe). Group III proteins represent WRKY domains with different zinc finger motifs (Cys2-His/Cys Cys-His2) (Eulgem et al., 2000; Finatto et al., 2018). The genomes of various plants have sequenced—presenting important knowledge about WRKY TFs and revealed that the WRKY TF family consists of a large number of genes (Zhang et al., 2011b; Xiong et al., 2013; Ayadi et al., 2016; Li et al., 2016a; Mohanta et al., 2016; Liu et al., 2017; Finatto et al., 2018) (**Table 1**). Plant-specific WRKY TFs, a major family of TFs, are a class of DNA-binding proteins found primarily in plants that have a variety of roles in plant processes, including growth, development, and stress signaling through autonomic and cross-regulation with TF and various other genes (Bakshi and Oelmüller, 2014). The first member of WRKY SPF1 superfamily was isolated from the sweet potato (*Ipomoea batatas*) (Ishiguro and Nakamura, 1994). In general, WRKY TF is expected to function as a key regulatory protein through precise binding to the W-box (TTGAC (C/T)) that regulates gene expression (Chi et al., 2013).

**Table 1:** Number of WRKY TFs genes in plants.

S. No	Name of plant	Number of WRKY TF gene
1	*A. thaliana*	74
2	*B. distachyon*	81
3	*C. sinensis*	51
4	*C. clementina*	48
5	*D. carota*	38
6	*G. max*	179
7	*J. curcas*	58
8	*M. esculenta*	117
9	*M. domestica*	123
10	*M. notabilis*	54
11	*O. sativa Indica*	116
12	*O. sativa japonica*	137
13	*P. vulgaris*	88
14	*P. trichocarpa*	119
15	*S. lycopersicum*	79
16	*S. tuberosum*	82
17	*V. vinifera*	98
18	*Z. mays*	180

**Figure 1:**
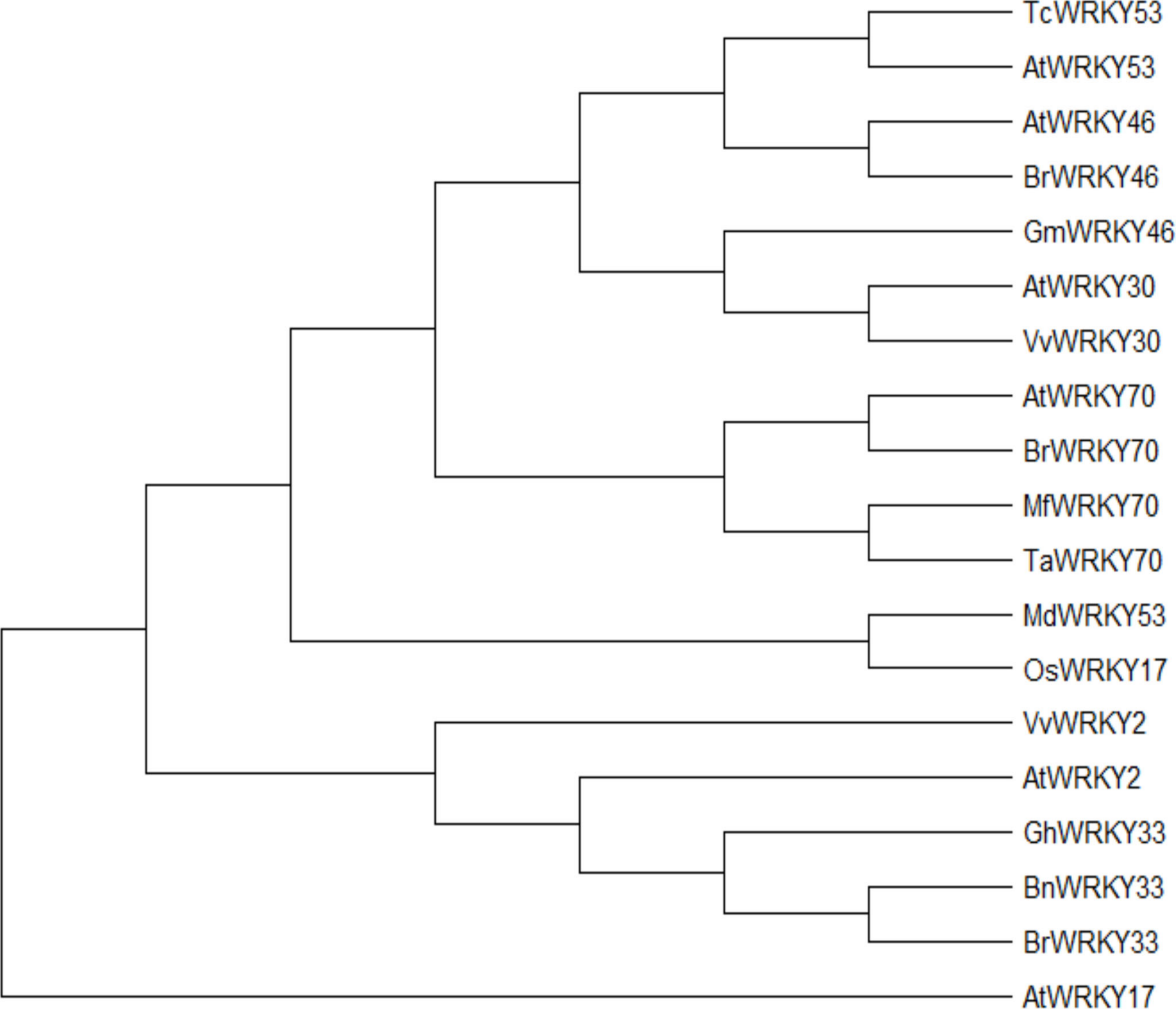
Neighbor-joining phylogeny of WRKY-related protein. MEGA X reconstructed the neighbor-joining phylogeny with 1000 bootstrap replicates and used maximum composite likelihood.

The coding sequence (CDS) of each WRKY gene was obtained from the National Center for Biotechnology Information (NCBI) (https://www.ncbi.nlm.nih.gov/) to build the phylogenetic tree using MEGA X and 1000 BS. It was shown in (**Figure 1**) that each homolog of WRKY genes showed the closest similarity, such as *AtWRKY53* with *TcWRKY53*, *AtWRKY46* with *BrWRKY46*, and *AtWRKY70*, *BrWRKY70*, *MfWRKY70* with *TaWRKY70*. As it was mentioned before that, *AtWRKY53* expression was induced by drought stress (Jiang et al., 2012). In contrast, *TcWRKY53* was induced by cold stress (Wei et al., 2008), illustrating that these two WRKY genes have a different role under different abiotic stress and species as well. It was also assumed that each WRKY gene might also contribute to multiple abiotic stresses.

## Drought stress-related WRKY TFs

**Figure 2:**
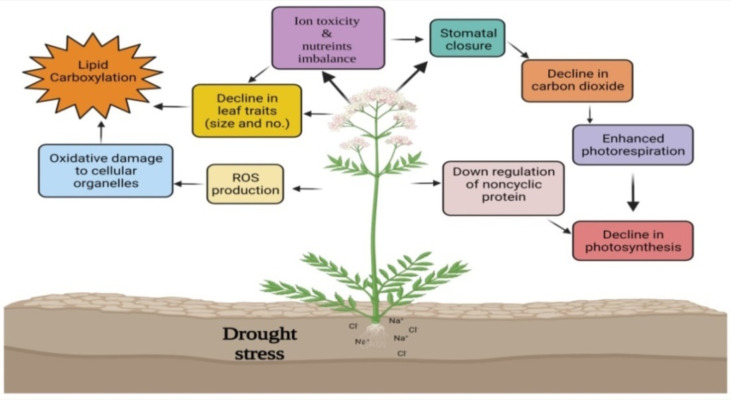
Effect of drought stress and the role of WRKY TFs in mitigating drought stress. Drought stress causes ROS production, oxidative damage, ion toxicity, and nutrient imbalance impairing plant growth and development. WRKY TFs regulate the expression of stress response genes and ROS scavenging enzymes. Overexpression of various WRKY TFs reduces ion loss and ROS accumulation, induces leaf stomatal mobility, decreases water loss rate thereby promote water retention, which overall improves phenotypic morphology and plant survival.

The expression of WRKY TF is induced when plants are exposed to various stresses or defense signals, including salicylic acid (SA) or other molecules. In addition to the fact that WRKY TF expression is rapid, transient, and tissue-specific, WRKY proteins also play diverse functions in plant defenses against different stresses including drought, plant growth, development, metabolism, trichome and embryonic morphogenesis, senescence, biosynthesis and regulation of hormonal signals (Wei et al., 2017) (**Figure 2**). The WRKY TFs present important roles in response and adaptation to drought stress (**Table 2**). Overexpression of *AtWRKY57* increased drought tolerance in *A. thaliana*. It has been studied that the Arabidopsis *WRKY57* transcription factor may confer drought tolerance to transgenic rice *O. sativa* plants. The overexpression of *AtWRKY57* in rice improved drought, salinity, and polyethylene gylcol (PEG) tolerance, indicating a possible role of *AtWRKY57* in crop development (Jiang et al., 2016). The *MaWRKY80* was up-regulated under drought stress conditions and was identified as a TF capable of binding to the W-box in *A. thaliana*. *MaWRKY80* overexpression exhibits improved phenotypic morphology, improved survival, lower water loss rate, and lower malondialdehyde (MDA) levels than WT (wild-type) under drought stress. Under drought stress, the transgenic *MaWRKY80*-leaves of *A. thaliana* showed lower reactive oxygen species (ROS) than WT. The *MaWRKY80* also promoted leaf stomata motility and water retention by regulating 9-cis-epoxycarotenoid dioxygenase (NCED) transcript and abscisic acid (ABA) biosynthesis in *A. thaliana* (Liu et al., 2020).

**Table 2:** Drought Stress-related WRKY TFs in plants.

S. No.	Gene	Species	Tolerance to stress	Reference
1	*AtWRKY53*	*A. thaliana*	drought	(Jiang et al., 2012)
2	*SlWRKY81*	*S. lycopersicum*	drought	(Ahammed et al., 2020)
3	*GhWRKY33*	*G. hirsutum L.*	drought	(Wang et al., 2019)
4	*SlWRKY72*	*S. lycopersicum*	drought	(Karkute et al., 2018)
5	*MuWRKY3*	*M. uniflorum lam.verdc.*	drought	(Kiranmai et al., 2018)
6	*TaWRKY2*	*T. aestivum L.*	drought	(Gao et al., 2018)
7	*TaWRKY1/33*	*T. aestivum L.*	drought	(He et al., 2016)
8	*ZmWRKY40*	*Z. mays*	drought	(Wang et al., 2018b)
9	*SbWRKY30*	*S. bicolor*	drought	(Yang et al., 2020)
10	*AtWRKY30*	*A. thaliana*	drought	(El-Esawi et al., 2019)
11	*VlWRKY48*	*CV.kyoho*	drought	(Zhao et al., 2018a)
12	*XsWRKY20*	*X. sorbifolium*	drought	(Xiong et al., 2020)
13	*GhWRKY41*	*G. hirsutum L.*	drought	(Chu et al., 2015)
14	*ZmWRKY106*	*Z. mays*	drought	(Wang et al., 2018a)
15	*VfWRKY1/2*	*V. faba L.*	drought	(Abid et al., 2017)
16	*AhWRKY*	*A. hypogaea L.*	drought	(Zhao et al., 2020b)
17	*VvWRKY13*	*V. vinifera L.*	drought	(Hou et al., 2020)
18	*BdWRKY36*	*B. distachyon*	drought	(Sun et al., 2015)
19	*GhWRKY27a*	*G. hirsutum*	drought	(Yan et al., 2015)
20	*MbWRKY1*	*M. baccata L.*	drought	(Han et al., 2018a)
21	*SpWRKY1*	*Phy. infestans*	drought	(Li et al., 2015)
22	*EjWRKY17*	*E. japonica*	drought	(Wang et al., 2021a)
23	*PheWRKY86*	*Phy. edulis*	drought	(Wu et al., 2022a)
24	*BoWRKY1o*	*B. oleracea* var. *A. DC*	drought	(Guo et al., 2021)
25	*TaWRKY46*	*T. aestivum L.*	drought	(Yu and Zhang, 2021)
26	*OsWRKY5*	*O. sativa*	drought	(Lim et al., 2021)
27	*CsWRKY26*	*C. sinensis*	drought	(Chen et al., 2021)

The sorghum WRKY TF, *SbWRKY30* primarily expressed in leaves and roots was induced *via* drought stress. In *A. thaliana* and rice, heterologous expression of *SbWRKY30* confers drought tolerance *via* disturbing root architecture. In addition, *SbWRKY30* induced *SbRD19* (a homologous gene of the drought stress response gene *RD19* in *A. thaliana*) expression in sorghum and the overexpression of *SbRD19* increased drought tolerance in Arabidopsis compared to WT plants. This suggests that *SbWRKY30* functions as a positive regulator in response to drought stress (Yang et al., 2020). Suppression of *GhWRKY21* has been shown to improve drought tolerance in cotton, although *GhWRK21* exhibits a negative role in drought response in cotton (Wang et al., 2021b). Overexpression of the *MuWRKY3* TF gene in peanuts (*A. hypogaea L*.) showed increased tolerance to drought stress and exhibited reduced and delayed wilting symptoms in transgenic plants than WT under drought stress imposition. This indicated that *MuWRKY3* (nuclear-localized) TFs controlled the expression of stress response genes and the actions of ROS scavenging enzymes, thereby led to increased drought tolerance in peanuts (Kiranmai et al., 2018). The expression analysis of *GhWRK25* revealed that *GhWRK25* gene is induced by biotic stress and several defense-related signaling molecules (Liu et al., 2016). Overexpression of *GhWRKY25* in *N. benthamiana* reduced plant tolerance to drought stress and increased tolerance to salt stress (Liu et al., 2016). The *GmWRKY12*, clustered in WRKYII, is 714 bp in length and encodes 237 amino acids. The *GmWRKY12* is expressed in various tissues, not only under normal conditions in soybean, but also strongly expressed under drought and salt treatments (Shi et al., 2018).

The *GhWRKY68* overexpression in *N. benthamiana*, a novel group of WRKY group IIC genes, responds to drought and salt stresses by regulating ABA signaling and modulating cellular ROS (Chi et al., 2013; Jia et al., 2019). The gene *BdWRKY36* belongs to the WRKY IIe group, designated from *B. distachyon*, the *BdWRKY36* localization in the nucleus is identified *via* the transient expression in onion epidermal cell. The C-terminal region of *BdWRKY36* was found to be transcriptionally active by transactivation assays in transgenic tobacco lines under drought stress. Overexpression of *BdWRKY36* resulted in less ion loss (IL) and ROS accumulation in tobacco lines. Whereas, under drought stress in *BdWRKY36*-overexpressing tobacco lines, the expression levels of ROS scavenging and stress response genes were up-regulated. Overall, *BdWRKY36* was found to act as a positive regulator of drought stress response through regulation of ROS homeostasis and regulation of transcription of stress-related genes (Sun et al., 2015; Li et al., 2020b). The *OsWRKY11* activates the drought-responsive gene transcription, namely *RAB21*, *via* binding directly to the promoter site, and the protein levels of *OsWRKY11* controlled by the system known as ubiquitin-proteasome (Lee et al., 2018; Liu et al., 2020). It was studied that *GmWRKY54* improved stomatal closure to reduce water loss, thus confirming drought tolerance in soybean through improved gene ontology (GO), co-expression network analysis, and physiological parameters. In transgenic soybean plants, expression of *GmWRKY54* confers drought tolerance by the constitutive promoter (*pCm*) and drought-induced promoter (*RD29a*). In soybean, the *GmWRKY54* activates genes (*PYL8, SRK2A, CIPK11*, and *CPK3*) by directly binding to the promoter region, and it has revealed that *GmWRKY54* played its function *via* ABA and Ca^2+^ signaling pathways. In transgenic Arabidopsis, *GmWRKY54* could also improve drought stress tolerance (He et al., 2016; Wei et al., 2019). The *GhWRKY59* plays an important role in regulating cotton’s response to drought. Studies have identified that key WRKY TFs are activated and phosphorylated by the MAP kinase cascade, which exhibited *GhMAP3K15*, *GhMKK4*, *GhMPK6*, *GhWRKY59*, and *GhDREB2*, as regulatory modules involved in regulating the response of cotton to drought (Li et al., 2017a).

Wheat (*Triticum aestivum*) is the main crop worldwide; its production in various areas is affected by drought. Therefore, improving the drought tolerance of wheat *via* breeding cultivars is an essential step for food security. It has been examined that *TaWRKY2* isolated from *T. aestivum* enhanced drought tolerance and increased grain productivity in wheat (Niu et al., 2012; Gao et al., 2018; El-Esawi et al., 2019). The WRKY30 TF, *AtWRKY30*, cloned from *A. thaliana*, overexpressed in wheat, which exhibited lower levels of hydrogen peroxide, electrolyte leakage, and malondialdehyde in transgenic plants compared to WT. Moreover, in transgenic wheat plants, some enzyme encoding stress-responsive genes (*WRKY19*, *TIP2, ERF5a*, *DREB1*, *DREB3*, and *AQP7*), were induced, which indicates *AtWRKY30* to be a possible candidate gene to improve stress-tolerance in wheat (El-Esawi et al., 2019). A WRKY TF, *GhWRKY33*, established in cotton, localizes to the cell nucleus and can bind to (W-box) cis-acting elements of target promoters. Moreover, *GhWRKY33* overexpression in Arabidopsis acts as negative regulator that mediates drought stress responses and contributes to ABA signaling (Wang et al., 2019; Khuman et al., 2020; Shaheen et al., 2020). It has been reported that the grape gene *WRKY48* is upregulated due to drought stress, fungal infection, and response to exogenous addition of plant hormones. In *A. thaliana*, over-expressed *V1WRKY48* form (cv. Kyoho), regulates a variety of drought stress responses and exhibits resistance to powdery mildew infection (Han et al., 2018c; Zhao et al., 2018a). The maize WRKY gene promoter region contains C-repeats, dehydration response element (DRE), cold response element (LTR), microbial biomass-C (MBC), and TCA elements that act on drought stress, flocculation, and SA. In transgenic Arabidopsis, the overexpression of *ZmWRKY106* (from the maize member WRKY group II) acted as a positive factor, which improved the drought and heat tolerance (Wang et al., 2018a; Hou et al., 2020). It has been recognized that the WRKY TF gene *ZmWRKY40*, is located in the core of mesophyll protoplasts and the promoter region of *ZmWRKY40* and has numerous transcriptional regulatory elements. A candidate gene, *ZmWRKY40*, improved drought tolerance in transgenic *A. thaliana* through regulation of stress-related genes under drought stress in transgenic lines where ROS levels decreased by enhancing the activity of two enzymes, peroxide dismutase (POD) and catalase (CAT) (Wang et al., 2018b; Leng and Zhao, 2020). The WRKY genes, *TaWRKY1* and *TaWRKY33* (group III and II) have reported to be localized in nucleus in wheat mesophyll protoplasts. In the promoter regions of these genes, several abiotic cis-acting elements were detected. Due to high temperature and ABA, *TaWRKY1* gene was up-regulated and down-regulated *via* low temperature. In addition, the *TaWRKY33* gene shows the higher response to ABA, jasmonic acid methyl ester, and to high and low temperatures. In Arabidopsis transgenic lines, *TaWRKY33* exhibited less water loss than the *TaWRKY1* gene, and the overexpressed *TaWRKY1* and *TaWRKY33* genes were associated in activation of various downstream stress-related genes, and higher germination rates under various stress conditions (He et al., 2016).

## Temperature stress-related WRKY TFs

Most plants grow in specific environments and repeatedly experience changes in external conditions. As a result, plants have evolved many complex mechanisms to resist various stresses. WRKY TFs are key proteins that respond to environmental stimuli by regulating gene expression (Xu et al., 2018; He et al., 2019). WRKY TFs are major plant-specific TFs that regulate numerous downstream stress response genes and play important roles in plant biotic and abiotic stress responses. Abiotic stressors, such as drought, heat, salinity, and cold are the main reasons why plants are undermining productivity around the world (Surendran et al., 2017). At the molecular level, WRKY-TFs are one of the most important families of plant-specific regulatory proteins in the plant kingdom, and are known to contribute to biotic and abiotic stress responses (Sarris et al., 2015; Joshi et al., 2016).

The high and low temperatures cause widespread agricultural damage, reducing crop yields and plant quality. To Protect plant cells from damage caused by extreme temperature changes essential for increasing agricultural production (Ohama et al., 2017). Due to global change, extremely high temperatures are getting a lot of attention and there is evidence that heat stress is responsible for biochemical changes in plants (Li et al., 2020b). Extremely high temperatures have become a major factor affecting plant growth, crop yield, fruit quality, flowering, plant biochemistry, morphology, and physiology (Goraya et al., 2017; Li et al., 2018). WRKY TF plays an important role in plant responses to heat stress. Most studies have shown that WRKY TF responds positively to plant tolerance to high temperatures. For example, in *A. thaliana* high-temperature treatment induces the expression of *AtWRKY25* and *AtWRKY26*, and inhibits *AtWRKY33*, whereas overexpression of *AtWRKY25/26* increases tolerance to heat stress in *A. thaliana* (Li et al., 2011). In peppers, *CaWRKY40* promotes stress resistance at high temperatures and the overexpression of *CaWRKY40* in tobacco reduces susceptibility to heat treatment, whereas loss of *CaWRKY40* reduces this tolerance (Liu et al., 2021). Inhibition of *AtWRKY41* expression in *A. thaliana* leads to reduced seed dormancy and suppression of high temperature (Chen et al., 2012; Ding et al., 2014). The overexpression of *TaWRKY33* in wheat enhances the high-temperature tolerance (El-Esawi et al., 2019). It has been studied that WRKY-TFs increase ROS production in the cell because of high-temperature stress in plants results in an excessive accumulation of ROS produced oxidative stress. Recent studies have shown that WRKY-TF is induced through ROS and contributes to the ROS elimination transformation pathway.

Oxidative stress is a severe stress caused by a variety of stresses, and ROS-mediated signaling is regulated by a delicate balance between production and clearance (Salvucci et al., 2001; Alvarez-Venegas et al., 2007). There are four types of reactive oxygen species in plants: oxygen, hydrogen peroxide, hydroxyl radicals, and superoxide anions. Several WRKY TFs (*WRKY6*, *WRKY30*, *WRKY22*, *WRKY8*, *WRKY53*, *WRKY48*, *WRKY39*, and *WRKY75*) are activated in *A. thaliana* in response to hydrogen peroxide treatment (Davletova et al., 2005; Jiang et al., 2017). It has been investigated that treatment of H_2_O_2_ activated higher expression of (*WRKY6*, *WRKY8*, *WRKY22*, *WRKY30*, *WRKY39*, *WRKY48*, *WRKY53*, and *WRKY75*) that could respond to a higher temperature in *A. thaliana* (Chen et al., 2010). *OsWRKY42* has been shown to play an important role as a negative regulator of oxidative stress, and overexpression of *OsWRKY42* in rice results in higher ROS accumulation (Han et al., 2014). Overexpression of *TaWRKY10* in wheat showed reduced malonaldehyde (MDA) accumulation, and low MDA was associated with a low rate of lipid peroxidation. This showed that the transgenic seedlings exhibited high resistance to oxidative stress due to increased expression of *TaWRKY10*, which resists reduced heat damage. The *AtWRKY28* was found to regulate the expression of downstream-associated genes through ROS in *A. thaliana* when exposed to oxidative stress (Niu et al., 2012; Babitha et al., 2013). The *ClWRKY20* belongs to group III of the WRKY family, and intracellular localization of *ClWRKY20* was found in the nucleus. The expression level of *ClWRKY20* was increased due to salinity, drought, and phytohormones (ABA, ET, and SA) treatment. *ClWRKY20* overexpression in transgenic Arabidopsis increased sensitivity to ABA at low temperatures, salinity, and during seed germination (Zhu et al., 2022). This study showed that WRKY-TF enhances plant tolerance to high temperature through transcriptional regulation (**Table 3**).

**Table 3:** Temperature stress-related WRKY TFs.

S. No.	Gene	Species	Tolerance to stress	References
1	*AtWRKY30*	*A. thaliana*	temperature	(El-Esawi et al., 2019)
2	*AtWRKY46*	*A. thaliana*	temperature	(Suzuki et al., 2005)
3	*OsWRKY77*	*O. Sativa*	temperature	(Lan et al., 2013)
4	*CaWRKY27*	*C. annuum*	temperature	(Dang et al., 2018)
5	*CaWRKY40*	*C. annuum*	temperature	(Dang et al., 2013)
6	*AtWRKY41*	*A. thaliana*	temperature	(Ding et al., 2014)
7	*TaWRKY70*	*T. aestivum*	temperature	(Wang et al., 2017)
8	*AtWRKY54*	*A. thaliana*	temperature	(Li et al., 2020b)
9	*PtWRKY13*	*P. tomentosa*	temperature	(Ren et al., 2019)
10	*PtWRKY50*	*P. tomentosa*	temperature	(Ren et al., 2019)
11	*ZmWRKY106*	*Z. mays*	temperature	(Wang et al., 2018a)
12	*AtWRKY39*	*A. thaliana*	temperature	(Li et al., 2010b)
13	*AtWRKY72*	*A. thaliana*	temperature	(Cheng et al., 2021)
14	*AtWRKY7*	*A. thaliana*	temperature	(Park et al., 2005)
15	*AtWRKY8*	*A. thaliana*	temperature	(Han et al., 2015)
16	*AtWRKY15*	*A. thaliana*	temperature	(Han et al., 2015)
17	*AtWRKY26*	*A. thaliana*	temperature	(Fu and Yu, 2010)
18	*AtWRKY33*	*A. thaliana*	temperature	(Fu and Yu, 2010)
19	*TaWRKY1*	*T. aestivum*	temperature	(Ren et al., 2019)
20	*NtWRKY6*	*N. tabacum*	temperature	(Macková et al., 2013)
21	*HaWRKY6*	*H. annuus*	temperature	(Giacomelli et al., 2012)
22	*ClWRKY20*	*C. lanatus*	temperature	(Zhu et al., 2022)

## Cold stress-related WRKY TFs

Cold stress (cold below 20°C and freezing below 0°C) adversely affects plant growth and development and greatly limits agricultural productivity. Plants adapt tolerance to cold stress, chilling and freezing by various physiological, protective, and molecular response systems. It has been studied *via* analyzing regulatory mechanism in plants, many genes have been identified that respond to cold stress at the transcriptional level (Ahmadizadeh and Heidari, 2014; Ritonga et al., 2021). Many WRKY TFs known to have important role in cold stress tolerance in various species (**Table 4**). Recent studies have shown that transgenic lines of Arabidopsis overexpressing *CsWRKY46* and cucumber WRKY show higher seedling viability when frozen at 4°C. In addition, the study identified transgenic *A. thaliana* in which overexpression of *GmWRKY21* (soybean WRKY) showed increased resistance to cold stress (Zhou et al., 2008; Zhang et al., 2016). Another study showed that *CsWRKY46* (belonging to the group II WRKY family) was localized in the nucleus, as determined by transient expression analysis. After freezing treatment, Arabidopsis lines, overexpressing *CsWRKY46*, *WRK46-OE1*, and *WRK46-OE5* had a higher survival rate than the WT. *CsWRKY46* confers cold tolerance to transgenic plants and modulates cold signaling pathways in an ABA-dependent manner. Whereas, overexpression of *OsWRKY76* was found to enhance cold stress tolerance at 4°C (Zhang et al., 2016). Overexpression lines compared to WT exhibited better surveillance under -20°C after 80 minutes and until 72 hours. The over-expressing plant lines had lower ion content leakage related to WT plants. From that, it could be assumed that overexpression lines could possess higher membrane stability (Yokotani et al., 2013).

**Table 4:** Cold stress-related WRKY TFs.

S. No.	Gene	Plant species	Factors	Responses	Reference
1	*AtWRKY34*	*A. thaliana*	Cold	Play a role as a negative regulator in cold stress	(Zou et al., 2010)
2	*VvWRKY24*	*V. vinifera*	Cold	Up-regulate regulation of hypothermia	(Wang et al., 2014b)
3	*OsWRKY76*	*O. sativa*	Cold	Tolerance to cold	(Yokotani et al., 2013)
4	*BcWRKY46*	*B. campestris*	Cold and Salt	Drought and salt tolerance	(Wang et al., 2012)
5	*VbWRKY32*	*V. bonariensis*	Cold	Tolerance to cold stress	(Wang et al., 2020)
6	*GmWRKY21*	*G. max*	Cold, Drought,	Tolerance to cold stress	(Zhou et al., 2008)
7	*VpWRKY2*	*V. pseudoreticulata*	Cold, ABA, and Salt	Tolerance to cold and salt stress	(Li et al., 2010a)
8	*TcWRKY53*	*T. caerulescens*	Cold, NaCl, and PEG	Play a role as a negative regulator in osmatic stress	(Wei et al., 2008)
9	*JrWRKY2*	*J. regia*	Cold and Drought	Cold and drought tolerance	(Yang et al., 2017)
10	*JrWRKY7*	*J. regia*	Cold and Drought	Tolerance to cold and drought stress	(Yang et al., 2017)
11	*LchiWRKY33*	*L. chinense* (*Lchi*)	Cold	Tolerance to cold stress	(Wu et al., 2022b)

## Salinity stress-related WRKY TFs

Soil salinity is one of the major abiotic stresses that affect the productivity of crops. Because the ionic and osmotic stresses of high salt concentrations in the soil affect the growth and development of plants. Salt stress is highly common in arid regions because of excessive evaporation leading to the accumulation of inorganic salts, which affects plant metabolism. With the success of traditional breeding approaches to improve stress-tolerant traits, transformation methods appear to be particularly beneficial for breeding stress-tolerant crops. In this regard, TFs play an important role as mediators in genetic engineering due to their unique roles in the regulation and modification of various stress-sensitive genes (Chaudhry et al., 2021; Hussain et al., 2021).

WRKY TFs also present a key role in salt stress response and tolerance (**Table 5**). Recent studies have shown that overexpression of *AtWRKY46* enhances root development during salt stress in Arabidopsis through modulation of ABA signaling. In addition, overexpression of *GhWRKY34* (*G. hirsutum*) enhances the plant’s ability to selectively absorb Na^+^ as well as K^+^ and maintain low Na^+^/K^+^ levels, thereby increasing resistance to salt stress in the leaves and roots of transgenic Arabidopsis plants (Dai et al., 2016). Overexpression of *GmWRKY54* (*WRKY soybean*) in transgenic Arabidopsis plants shows salt tolerance, it has indicated that WT plants showed 25% survival while over-expressing lines showed 70% survival under 180 mM NaCl treatment (Zhou et al., 2008). Another study found that N. benthamiana *GmWRKY17* (cotton WRKY) improved salinity stress tolerance as measured by physiological analyzes of germination rate, root growth, survival, and leaf water loss (Yan et al., 2014). A new WRKY gene was isolated from *M. xiaojinensis*, namely *MxWRKY55*, and it is localized in the nucleus. The expression level of *MxWRKY55* in *M. xiaojinensis* seedlings was affected by salinity, low Fe, and high Fe stresses, and *MxWRKY55* also increased salinity and iron tolerance when introduced into *A. thaliana*. Overexpression of *MxWRKY55* in *A. thaliana* showed high levels of chlorophyll and proline, as well as increased activity of superoxide dismutase (SOD), peroxidase (POD), and catalase (CAT). Similarly, *MxWRKY55* in *A. thaliana* resulted in lower levels of malondialdehyde (MAD), particularly in response to salt stress. In addition, overexpression of *MxWRKY55* in transgenic *A. thaliana* showed greater root length, mass, chlorophyll, and iron content compared to WT (Han et al., 2020). Based on these properties, it has been demonstrated that *MxWRKY55* can play a positive role in the process of salt resistance, resistance to high Fe, and low Fe content. Another study showed that the growth and development of *M. xiaojinesis* (semi-dwarf apple in China) was affected by the salinity and Fe. The novel WRKY *MxWRKY53/64* gene isolated from *M. xiaojinesis* is a nuclear-localized protein and its expression level is strongly influenced by salt as well as Fe, when *MxWRKY53/64* was introduced into transgenic *A. thaliana*, resistance to salinity and iron stress was significantly increased (Han et al., 2021a; Han et al., 2021b). Moreover, the over-expression of wheat WRKY TF, the *TaWRKY93* in *A. thaliana* showed high salt tolerance, low temperature, and osmotic stress tolerance (Qin et al., 2015).

**Table 5:** Salinity stress-related WRKY TFs.

S. No.	Gene	Plant	Tolerance to stress	Reference
**1**	*FcWRKY70*	*F. crassifolia*	Salt	(Wang et al., 2007)
**2**	*GmWRKY17*	*G. max*	Salt	(Yan et al., 2014)
**3**	*ZmWRKY17*	*Z. mays*	Salt	(Cai et al., 2017)
**4**	*SbWRKY30*	*S. bicolor*	Salt	(Yang et al., 2020)
**5**	*GbWRKY1*	*G. barbadense*	Salt	(Luo et al., 2020)
**6**	*IbWRKY47*	*I. batatas*	Salt	(Qin et al., 2020)
**7**	*PgWRKY33/62*	*P. glaucum*	Salt	(Chanwala et al., 2020)
**8**	*SbWRKY50*	*S. bicolor*	Salt	(Song et al., 2020b)
**9**	*VpWRKY1*	*V. pseudoreticulata*	Salt	(Li et al., 2010a)
**10**	*VpWRKY2*	*V. pseudoreticulata*	Salt	(Li et al., 2010a)
**11**	*MbWRKY5*	*M. baccata*	Salt	(Han et al., 2018b)
**12**	*CmWRKY*	*C. pepo*	Salt	(Bankaji et al., 2019)
**13**	*PbWRKY40*	*P. betulaefolia*	Salt	(Lin et al., 2022)
**14**	*ClWRKY20*	*C. Lanatus*	Salt	(Zhu et al., 2022)
**15**	*MxWRKY53*	*M. xiaojinensis*	Salt	(Han et al., 2021b)
**16**	*MxWRKY64*	*M. xiaojinensis*	Salt	(Han et al., 2021a)
**17**	*AhWRKY75*	*A. hypogaea L.*	Salt	(Zhu et al., 2021)
**18**	*MfWRKY70*	*M. Flabellifolia*	Salt	(Xiang et al., 2021)

## WRKY TFs as key regulators in plant growth and development

The WRKY TF is one of the largest TF families in plants, which in addition to stress response and defense regulation significantly contributes to plant growth and development. Various WRKY genes have been reported in different plant species that promote growth and development (Zhang et al., 2017) (**Table 6**). The *AtWRKY28* gene, *AtWRKY2*, and *AtWRKY34* which are involved in macrospore fate, pollen tube extension, pollen production, seed germination, and early growth after germination. *AtWRKY2* (a knockout mutant exhibiting high sensitivity to ABA) plays an important role in seed germination (Jiang and Yu, 2009). The overexpression of *VvWRKY30* in Arabidopsis increased resistance to salt stress at various growth stages by regulating ROS clearance and osmotic accumulation (Zhu et al., 2019). In soybean, *GmWRKY12* induced a positive role in ABA, salt, and drought stresses (Shi et al., 2018).

**Table 6:** Role of WRKY TFs in plant growth and development.

S. No.	Name	Plant	Function	References
**1**	*VvWRKY30*	*V. vinifera*	Increasing salt stress resistance by ROS and accumulation of osmoticum.	(Zhu et al., 2019)
**2**	*GmWRKY12*	*G. max*	Drought and salinity tolerance	(Shi et al., 2018; Zhang et al., 2020)
**3**	*MdWRKY40*	*M. domestica*	Important regulators of wound-induced anthocyanin biosynthesis	(An et al., 2019)
**4**	*TaWRKY51*	*T. aestivum L.*	Promotes lateral root formation due to negative regulation of ethylene biosynthesis	(Hu et al., 2018)
**5**	*GhWRKY59*	*G. hirsutum*	Drought responses	(Li et al., 2017a)
**6**	*HbWRKY82*	*H. brasiliensis*	Abiotic resistance and leaf aging	(Kang et al., 2021)
**7**	*MfWRKY70*	*M. Flabellifolia*	Drought and salinity tolerance	(Xiang et al., 2021)
**8**	*HmoWRKY40*	*H. monacanthus*	Betalain biosynthesis	(Zhang et al., 2021b)
**9**	*MxWRKY64*	*M. xiaojinensis*	It plays an important role in response to Fe and salt stress	(Han et al., 2021a)
**10**	*AhWRKY75*	*A. hypogaea L*	Conferred salt tolerance in transgenic peanut lines	(Zhu et al., 2021)
**11**	*BoWRKY10*	*B. oleracea* var.*acephala DC*	Regulation of drought stress tolerance	(Guo et al., 2021)
**12**	*AtWRKY28*	*A. thaliana*	Oocyte development	(Zhao et al., 2018b)
**13**	*AtWRKY2*	*A. thaliana*	Seed germination, growth after germination	(Jiang and Yu, 2009)
**14**	*AtWRKY10*	*A. thaliana*	The size of the seed	(Luo et al., 2005)
**15**	*AtWRKY34*	*A. thaliana*	Seed germination, growth after germination	(Guan et al., 2014)
**16**	*AtWRKY41*	*A. thaliana*	The dormancy of seed	(Ding et al., 2014)
**17**	*AtWRKY44*	*A. thaliana*	In the proanthocy seed coat of tannins	(Gonzalez et al., 2016)
**18**	*OsWRKY78*	*O. sativa*	The development of seed and stem elongation	(Zhang et al., 2011a)
**19**	*OsWRKY24*	*O. sativa*	Increased lamina inclination and grain size through cell elongation.	(Jang and Li, 2018)
**20**	*GhWRKY42*	*G. hirsutum*	Premature leaf senescence and stem development	(Gu et al., 2018)
**21**	*AtWRKY23*	*A. thaliana*	Root growth and biosynthesis of flavanols	(Grunewald et al., 2012)
**22**	*GhWRKY91*	*G. hirsutum*	Leaf senescence and stress response	(Gu et al., 2019b)
**23**	*OsWRKY93*	*O. sativa*	Leaf senescence and in response to fungi attack	(Li et al., 2021)
**24**	*BrWRKY6*	*B. rapa ssp.pekinensis*	Leaf senescence	(Fan et al., 2018)
**25**	*GhWRKY27*	*G. hirsutum*	Leaf senescence	(Gu et al., 2019a)
**26**	*PyMYB114*	*Red-Skinned pears*	Regulate anthocyanin biosynthesis and transport	(Li et al., 2020a)
**27**	*WRKY6*	*A. thaliana*	Improve FA accumulation and seed yield	(Song et al., 2020a)
**28**	*TaWRKY40-D*	*T. aestivum L.*	Association to the promotion of leaf senescence with jasmonic acid and abscisic acid	(Zhao et al., 2020a)
**29**	*WRKY46/6*	*A. thaliana*	PBZ/SA-mediated leaf senescence	(Zhang et al., 2021a)
**30**	*WRKY45*	*A. thaliana*	Positive regulator of age-triggered leaf senescence	(Chen et al., 2017)
**31**	*VvWRKY2*	*V. venifera*	Vigor, yield, and tuber quality	(Chiab, 2021)
**32**	*AtWRKY26*	*A. thaliana*	Leaf senescence	(Li et al., 2017b)
**33**	*WRKY12/13*	*A. thaliana*	Regulate flowering time	(Li et al., 2016b)
**34**	*WRKY42*	*A. thaliana*	Root hair growth and development	(Moison et al., 2021)
**35**	*OsWRKY11*	*O. sativa*	Flowering time and plant height	(Cai et al., 2014)
**36**	*AtWRKY45*	*A. thaliana*	Play a key role in Phosphate uptake	(Wang et al., 2014a)
**37**	*AtWRKY42*	*A. thaliana*	Play a great role in phosphate uptake	(Su et al., 2015)
**38**	AtWRKY71	*A. thaliana*	Flowering time	(Yu et al., 2016)
**39**	*MxWRKY55*	*M. xiaojinensis*	Tolerance to salt, low-iron and high-iron stress	(Han et al., 2020)

There are several WRKY genes involved in plant root development. The *TaWRKY51*, an important WRKY TF that increases lateral root formation through the regulation of ethylene biosynthesis in wheat (Hu et al., 2018). The study also reported that *TaWRKY51* regulates lateral root formation *via* the ethylene and auxin signaling pathways (Hu et al., 2018). *AtWRKY23* expression induced by the auxin response factor7 (ARF7) and auxin response factor 19 (ARF19) (serve as part of the auxin feedback loop), help to regulate the growth of plant roots and the synthesis of flavonoids (Grunewald et al., 2012). Both *AtWRKY75* and *AtWRKY44* are involved in root hair development. *AtWRKY44* is also a downstream gene (*TTG1* and *GLABROUS1*) expressed in root hairs that act jiontly with *GLABRA2* to regulate root hair growth in plants (Johnson et al., 2002). Studies have shown that the number and length of root hairs are increased in *AtWRKY75* (Knockout mutant) compared to the WT, suggesting that *AtWRKY75* is a negative regulator of root hair development (Devaiah et al., 2007).

A novel WRKY TF, designated *HbWRKY82*, was identified based on stress-related WRKY in rubber trees, encoded by nuclear proteins and present an important function as a transcriptional activator. Exogenous ethrel and ABA stimulation induce *HbWRK82* transcriptional activity, which play important roles as transcriptional regulators in ethrel and in response to ABA-mediated leaf senescence and abiotic stress (Kang et al., 2021). The *WRKY70* is involved in biological stress as a positive regulator and has a negative role in abiotic stress signaling in Arabidopsis and several other plant species. The localization of *MfWRKY70* in the nucleus was confirmed by examining *MfWRK70* from *M. flabellifolia* in *Arabidopsis* model plants. The *MfWRKY70* is reported to have an essential role in drought, osmotic pressure, and salinity tolerance by promoting root growth and water retention. Under stress conditions, *MfWRKY70* enhanced the antioxidant enzyme system, maintaining ROS homeostasis and stability of membrane lipids (Xiang et al., 2021).

A novel WRKY TF, the *HmoWRKY40* was identified from the transcriptomic data of pitaya (*H. monacanthus*), and the *HmoWRKY40* transcriptionally activates *HmoCYP76AD*, which regulates ptiaya betalain biosynthesis (Zhang et al., 2021b). Fe and high salinity affect the growth and development of *M. xiaojiensis*, a semi-dwarf apple in China. The newly isolated WRKY gene from *M. xiaojinesis*, namely *MxWRKY64* (localization in the nucleus)was introduced into *A. thaliana*, which showed increased resistance to Fe and salts, and overexpression of *MxWRKY64* in transgenic *A. thaliana* under Fe stress resulted in higher levels of mass, root length, chlorophyll, and Fe content compared to WT (Han et al., 2021a). A novel WRKY-TF gene *AhWRKY75* (WRKYIIC) identified from M34 (salt-tolerant mutant) confers salt tolerance to transgenic peanut strains by increasing the efficiency of ROS removal system and photosynthesis during stress treatment (Zhu et al., 2021). In flowering plants, female gonadal megasporoblasts (MMCs) start as single cells in each ovule, and Arabidopsis cytochrome P450 (KLU) functions through the SWR1 chromatin remodeling complex to promote *WRKY28* expression in oocyte primordial (Zhao et al., 2018b). The studies have suggested that WRKY genes play a key role in seed germination and post-germination growth. The Arabidopsis *WRKY2* TF is involved in seed germination and post-emergence stunting (Jiang and Yu, 2009), plant (male) gamete formation with complex and dynamic changes in gene expression. Studies have shown that WRKY2 and its close homolog WRKY34 (pollen-specific) TFs participated in male gametogenesis in *A. thaliana* (Guan et al., 2014).

**Figure 3:**
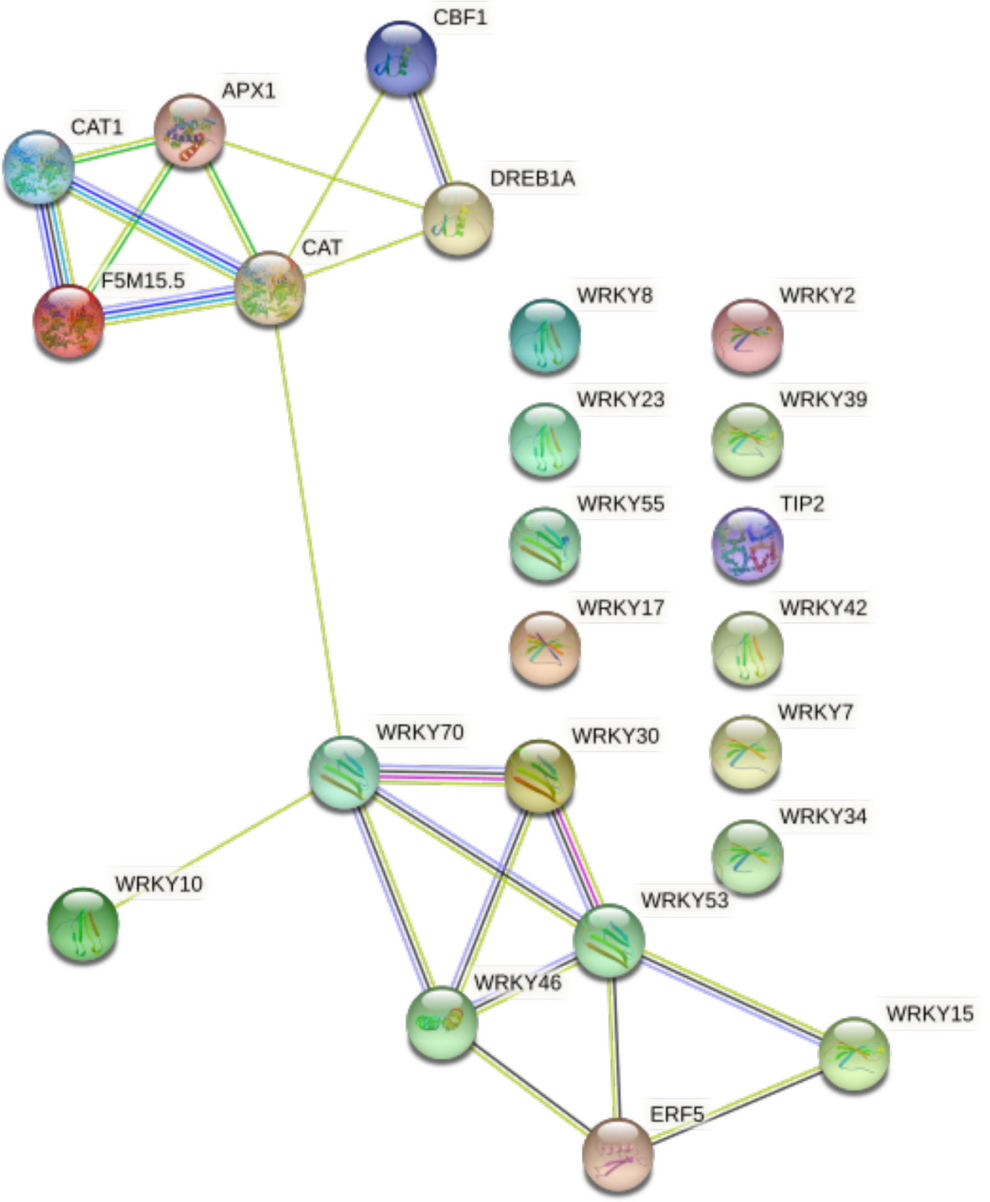
The interaction network analysis of some WRKY genes identified in *Arabidopsis thaliana* using STRING: Line colors are associated with interaction types. Around the green line gene, pink lines are experimentally determined, black lines mean co-expression, dark blue lines mean gene co-occurrence, and blue lines mean protein homology.

Interaction of WRKY genes with some stress-related genes to improve plant abiotic stress tolerance in plants was shown in **Figure 3**. The interaction network with STRING (https://string-db.org/cgi/) was recognized. The result showed that several WRKY genes correlate with abiotic stress-related genes; for instance, the above mentioned *AtWRKY30* cloned TFs from Arabidopsis; its over-expression in wheat showed improved stress tolerance. Moreover, in transgenic wheat, antioxidant genes such as APX1, CAT, CAT1, F5M15.5, ERF5, CBF1, and DREBIA play key roles as stress-responsive genes (El-Esawi et al., 2019). It was speculated that correlated genes might have a positive or negative correlation in response to abiotic stress.

## Conclusion and future prospects

**Figure 4:**
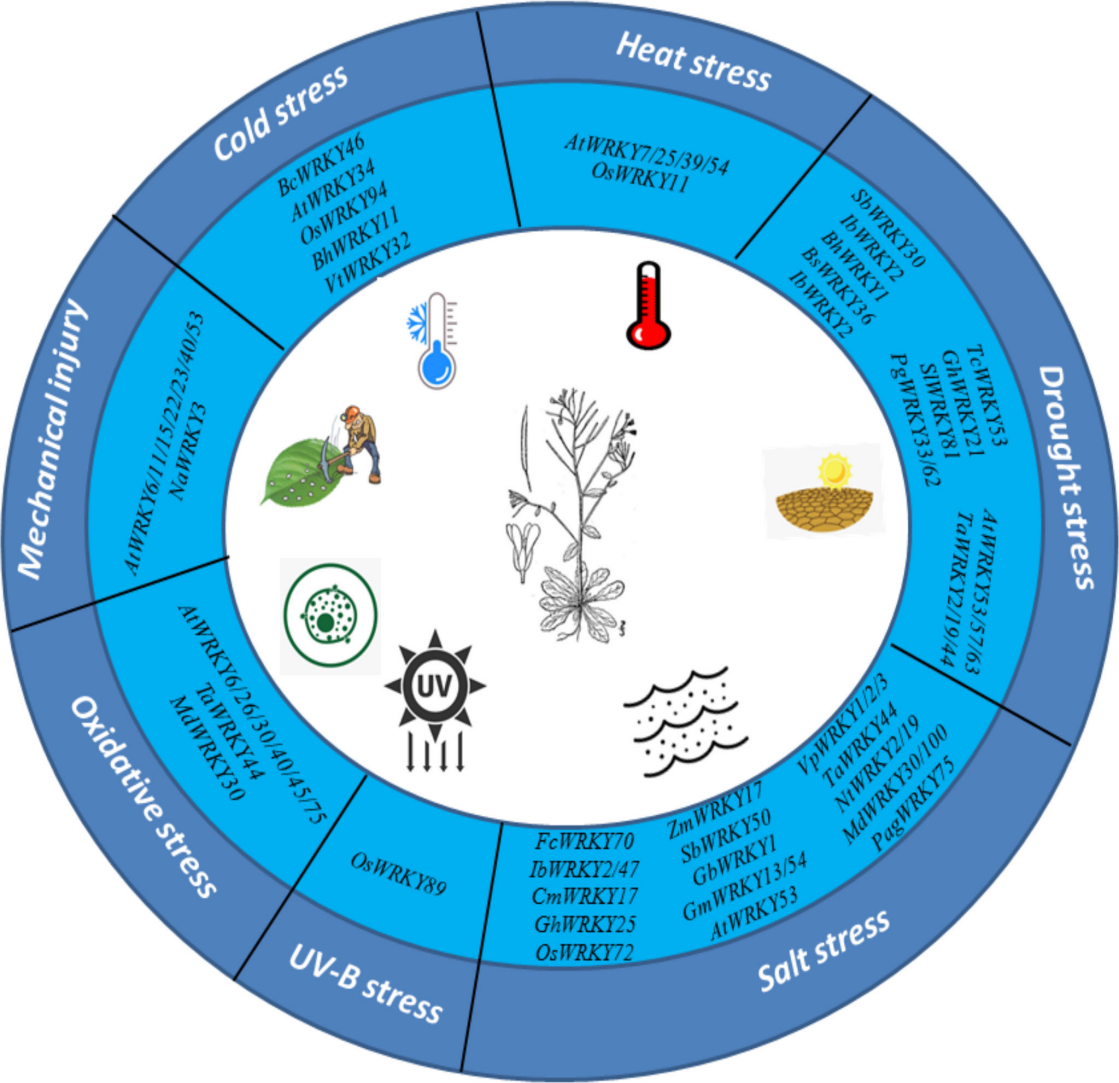
WRKY-TFs response to various abiotic stresses.

Plants are considered as sessile organisms that cannot avoid adverse abiotic stresses as well as other major environmental stresses and have developed complex signaling networks composed of multiple pathways. One of the largest TF families, WRKY-TFs act as molecular switches that regulates the expression of stress-sensitive genes. Stress-induced WRKY-TF expression is regulated by a complex transcriptional regulatory network that allows plants to maintain the proper balance between growth and stress response. This review discusses the recent studies of WRKY-TF. Many studies have shown that WRKY-TFs play important roles in abiotic stress tolerance (**Figure 4**). Nowadays the sequencing of plant genomes has increased largely; especially in economically important crops and whole-genome identification of the WRKY gene (with respect to functional plant genes) facilitate screening. Previous studies have demonstrated that the WRKY gene primarily depends on its functional assumptions and transcriptome. In addition, genetic confirmations joined to the latest technologies are increasing to confirm the novel role of the WRKY genes, expression of WRKY-TF or downstream genes regulated by self-regulation of WRKY-TF, which helps to simplify the regulatory network of responses to abiotic stresses. Future studies should explore noncoding RNAs and epigenetic modifications involved in the regulation of WRKY-TFs. Based on current studies the role of WRKY-TFs in regulating plant responses related to abiotic stresses, particularly drought, salinity, and temperature stress, are not sufficiently detailed, particularly at the transcriptional level. Finally, the use of WRKY-TF screening for plant stress tolerance in context to increase climate change significantly improves crop yield and crop quality.

## Author contributions

MK, and AH planned and designed this review manuscript. MK, AH, and HM wrote this review paper. FR, QA, MC, QM, MA, WZ, RMA, and RB helped to improve the manuscript writing. FL and HM contributed to the critically revising of the manuscript. All the authors have reviewed, edited, and approved the manuscript before submission.

## Funding

This work was supported by grant from the Lushan Botanical Garden, Chinese Academy of Sciences (No. 2021ZWZX28 to HM), and by grant from the National Natural Science Foundation of China (32100297 to FL).

## Conflict of interest

The authors declare that the research was conducted in the absence of any commercial or financial relationships that could be construed as a potential conflict of interest.

## Publisher’s note

All claims expressed in this article are solely those of the authors and do not necessarily represent those of their affiliated organizations, or those of the publisher, the editors and the reviewers. Any product that may be evaluated in this article, or claim that may be made by its manufacturer, is not guaranteed or endorsed by the publisher.
